# Correlation analysis between heart rate variability, epicardial fat thickness, visfatin and AF recurrence post radiofrequency ablation

**DOI:** 10.1186/s12872-022-02496-x

**Published:** 2022-02-22

**Authors:** Bin Jian, Zhen Li, Jianing Wang, Chuanlin Zhang

**Affiliations:** Department of Cardiology, Renmin Hospital, Hubei University of Medicine, No.39 Chaoyang Middle Road, Shiyan, 442000 Hubei China

**Keywords:** Heart rate variability, Epicardial fat thickness, Visfatin, Atrial fibrillation recurrence, Post radiofrequency ablation

## Abstract

**Background:**

The aim of the present study is to investigate the possible correlation between heart rate variability (HRV), epicardial fat thickness (EFT), visfatin and AF recurrence post radiofrequency ablation.

**Methods:**

Data of 337 AF patients to whom radiofrequency ablation therapy had been initiated at our hospital over the past three years were evaluated. The patients enrolled were divided into the non-recurrence group (102 patients) and the recurrence group (235 patients) according to AF recurrence in the preceding 12 months. General data in the two groups were collected and HRV, EFT, and visfatin levels were comprehensively compared for each patients of the two groups.

**Results:**

The recurrence group showed significantly higher results in rMSSD, PNN50, HF, total EFT, and visfatin but with evidently lower results in LF/HF when comparing the non-recurrence group (*P* < 0.05). The significantly different general variables in the general data and laboratory parameters, rMSSD, PNN50, HF, total EFT, visfatin, LF/HF were used as independent variables, and AF recurrence post radiofrequency ablation was used as dependent variables. Logistic regression analysis revealed that the risk factors of AF recurrence post radiofrequency ablation were rMSSD, PNN50, HF, total EFT, visfatin, and LF/HF, and the difference was statistically significant (*P* < 0.05).

**Conclusion:**

HRV, EFT, visfatin appear to show high association with AF recurrence post radiofrequency ablation.

## Background

Atrial fibrillation is one of the most common cardiac arrhythmias hat may occur at any age. The overall incidence of AF to be 2.8%-15.8% in the general population with a trend towards a gradual increase with age annually [1]. AF may cause embolic events, increase the risk of death and reduced quality of life [2]. Prompt management of AF is thus imperative. Despite that catheter radiofrequency ablation has been generally applied for the treatment of AF clinically, the rate of postprocedural AF recurrence is still high (34.0%) [3]. Therefore, it is important to determine the markers that may effectively detect recurrence post catheter radiofrequency ablation. Heart rate variability (HRV) refers to the minute change of time between successive cardiac cycles, namely the degree of sinus arrhythmia, which is an important method for quantitative analysis of autonomic nervous function at present. HRV index includes time domain index and frequency domain index. Among the time domain index, standard deviation of the normal-to-normal sinus-initiated interbeat-intervals (SDNN) reflects the total activity of sympathetic and parasympathetic nerves; 24-h standard deviation of the average normal-to-normal sinus-initiated interbeat-intervals (SDANN) reflects sympathetic tension. root mean square standard deviation of adjacent R-R intervals (rMSSD) reflects parasympathetic tone changes; the percentage of adjacent NN intervals that differed from each other by more than 50 ms (PNN50) reflects parasympathetic tension activity. Among the frequency domain index, low frequency (LF) mainly reflects sympathetic nerve activity; high frequency (HF) reflects vagus nerve activity; LF/HF reflects the relationship between sympathetic and vagal nerve balance [4]. The dysfunction of the autonomic nervous system may trigger and maintain AF by increasing the heterogeneity of electric activities of the atrium. Previous studies showed that the HRV was low in most patients after radiofrequency ablation than the preprocedural level, indicating that the autonomic effect was one of the mechanisms in the treatment of atrial fibrillation [5–7].

However, the postprocedural HRV is notably lower than that before radiofrequency ablation and the relation between normal and abnormal HRV and AF recurrence remains unclear [8]. Epicardial fat thickness (EFT) can secrete anti-inflammatory and anti-atherosclerotic factors under physiological conditions, but under pathological conditions, the disequilibrium between anti-inflammatory factors and anti-atherosclerotic factors secreted by EFT may trigger the onset and development of cardiovascular disease [9]. The potential correlation between EFT and AF recurrence post radiofrequency ablation remains debatable. Visfatin is a lipid factor secreted by the adipose tissues in the internal organs and is involved in fibrosis and inflammatory reaction and is intimately linked to many inflammation-mediated diseases [10]. However, limited studies exist on visfatin and AF recurrence post radiofrequency ablation so far. Understanding the influencing factors of atrial fibrillation recurrence may offer insight for clinical development and guide medical treatment. Based on this, this study was conducted aimed at exploring the possible correlation between HRV, EFT, and visfatin and AF recurrence post radiofrequency ablation.

## Methods

### Clinical data

Patients who received radiofrequency ablation at our hospital over the past three years were included in this study. The patients were divided into the non-recurrence group (102 patients) and the recurrence group (235 patients) according to whether the results of postoperative review suggested AF recurrence in the preceding 12 months. Inclusion criteria: patients consented to radiofrequency ablation therapy and had recurrence post radiofrequency ablation; patients had no cardiac valvular disease and were not contraindicated for anticoagulation; patients had no history of prior surgery. Exclusion criteria: concomitant thrombus of the left atrium, receipt of pacemaker implantation post non-first radiofrequency ablation, hyperthyroidism-induced AF, pregnant or lactating women, acute coronary syndrome, severe hepatic or renal failure or disorder, acute or chronic infection, concurrent malignancy, and receipt of antibiotics or surgical treatment within the preceding months. A total of 388 patients underwent radiofrequency ablation during 3 years. Fifty-one patients were excluded due to lack of laboratory tests and postoperative echocardiography. Finally, 337 patients with atrial fibrillation who underwent radiofrequency ablation were included in the study. Our study was approved by the Ethic Committee of Renmin Hospital, Hubei University of Medicine (RMH-2072302)and all patients provided written informed consent. The study protocol was in accordance with Helsinki Declaration.

### General data

Data on gender, age, smoking, body mass index (BMI), coronary heart disease, cardiac insufficiency and medications were obtained from the patients.

#### HRV

The Holter recording was carried out by 12 lead synchronous dynamic electrocardiogram system (Century Golden Co). The Holter results and HRV indexes of the patients were recorded before and 2 to 3 days after operation. SDNN reflects various frequency components of 24 h heart rate variation and evaluates the total tension of sympathetic and parasympathetic nerves; SDANN reflects the ultra-low frequency component of heart rate variability, evaluates the sensitive index of sympathetic nerve function, and reflects the slow change of heart rate; rMSSD reflects the ultra-high frequency composition of heart rate variability and is a sensitive indicator of parasympathetic function, reflecting the rapid change of heart rate; PNN50 reflects the beat to beat variation of cardiac cycle, evaluates parasympathetic sensitivity index, and reflects the rapid change of heart rate. LF is affected by both sympathetic nerve and vagus nerve; HF reflects vagal nerve activity. LF/HF reflects the balance between plant nerves, and its increase indicates sympathetic nerve excitation, which can be used to evaluate the physiological variation of sympathetic and parasympathetic nerve tension in an individual, or specifically analyze the damage of a single autonomic nerve component in a patient [4].

#### EFT

The volume of EFT was measured using 65-slice helical CT (Toshiba and Germany). First, the patients were infused with 370 mg non-ionic contrast iopamidol at a flow rate of 5.0 ml/s, and 50 ml normal saline at the same rate simultaneously. The slice thickness was 0.5 mm, tube voltage 120 kV, tube self-adjusting current (upper limit 200 m), and tube rotation speed 0.33 s/r. Using the contrast tracking method, the region of interest (ROI) was chosen at the plane of the aortic root for measurement of CT value, and dynamic same plane monitoring was triggered after injection of contrast agent and when the CT value of the ROI exceeded, scan was triggered after 6 s delay. Patients were asked to hold breath during the scan. The region scan ranged from the carina of the trachea to the cardiac apex. A designated workstation was used to reconstruct EAT images at a thickness of 0.75 mm. The epicardial border was manually delineated by experienced radiologists using Volumer layer by layer and the CT value of the adipose tissue was set at -50 to -200 HU, the total epicardial fat volume was automatically calculated. Thereafter, the epicardial fat adjacent to the left ventricle anterior to the mitral annulus, the epicardial fat adjacent to the right atrium anterior to the right superior pulmonary vein and the epicardial fat below the plane of the coronary sinus were manually removed from the total epicardial fat, and the remaining epicardial fat was the epicardial fat volume around the left atrium.

### Visfatin measurement

Five to eight mL venous blood was obtained from patients after overnight fast. The supernatant was collected after centrifugation and visfatin levels were determined using double antibody sandwich ELISA of commercial kits from Jingmei Biological Co.

### Criteria for determination of AF recurrence

Radiofrequency ablation was performed under the guidance of a 3D navigation system. The end point of surgery was the complete electric isolation of the pulmonary vein. ECG examinations were performed preoperatively and at 1, 3, 6, 9, and 12 months postoperatively, with 12-lead ECG and 24 h Holter. Atrial fibrillation recurrence was defined as: AF lasted for ≥ 30 s on 12-lead ECG or 24 h Holter after ≥ 3 months of follow-up post radiofrequency ablation, atrial flutter or atrial tachycardia was demonstrated [11]. All electrocardiograms were analyzed and interpreted by skilled electrocardiologists.

### Statistical analysis

Data analysis was conducted using SPSS 21.0 and database was established using Excel. Normally distributed count data were expressed as mean ± SD, and univariate analysis of variance was used for comparison between groups and LSD test was used for intragroup comparison. Categorical data was expressed in rate (%) and compared using χ^2^ test. Pearson linear correlation analysis was performed to identify the correlation between HRV, EFT, and visfatin and AF recurrence post radiofrequency ablation. Multivariate Logisitic regression was used to analyze the influencing factors of atrial fibrillation recurrence after radiofrequency ablation. A *P* < 0.05 indicates significant statistical difference.

## Results

### Comparison of general data between the two groups

In non-recurrence group, there were 55 males and 47 females. The age ranged from 43 to 67 years, with a mean age of (55.29 ± 12.01) years. In recurrence group, there were 125 males and 110 females. The age ranged from 44 to 68 years, with a mean age of (55.12 ± 12.09) years. There were no statistical differences in gender, age, BMI, smoking, coronary heart disease, diabetes, cardiac insufficiency, dyslipidemia, ACEI/ARB, β receptor blockers, anticoagulants, statins (*P* > 0.05) (Table 1).

**Table 1:** Comparison of general data between the two groups

Variables	The recurrence group (n = 235)	The non-recurrence group (n = 102)	χ^2^/t	P
Gender (male/female) (n)	125/110	55/47	0.015	0.902
Age (years)	55.12 ± 12.09	55.29 ± 12.01	-0.119	0.905
BMI (kg/m^2^)	25.32 ± 2.19	25.08 ± 2.13	0.932	0.352
Smoking (n)	35	13	0.269	0.604
Coronary heart disease (n)	52	14	3.188	0.074
Diabetes (n)	38	15	0.115	0.734
Cardiac insufficiency (n)	73	27	0.719	0.396
Dyslipidemia (n)	103	34	3.248	0.072
*Medications*				
ACEI/ARB (n)	89	34	0.632	0.427
β receptor blockers (n)	167	82	3.208	0.073
Anticoagulants (n)	162	78	1.970	0.160
Statins (n)	97	34	1.889	0.169

BMI, body mass index; ACEI, angiotensin-converting enzyme inhibitors; ARB, angiotensin receptor blockers

#### Comparison of HRV, EFT, and visfatin levels between the two groups

There was no statistical difference in SDNN and LF between the two groups (*P* > 0.05). The recurrence group showed significantly higher results in rMSSD, PNN50, HF, total EFT, and visfatin but with evidently lower results in LF/HF when comparing the non-recurrence group (*P* < 0.05) (Table 2).

**Table 2:** Comparison of HRV, EFT, and visfatin levels between the two groups

Variables	The recurrence group (n = 235)	The non-recurrence group (n = 102)	t	*P*
*HRV*				
SDNN (ms)	101.04 ± 14.29	100.01 ± 13.18	0.622	0.534
rMSSD (ms)	25.23 ± 2.12	21.76 ± 2.31	13.430	< 0.001
PNN50 (%)	7.31 ± 1.23	4.67 ± 1.04	20.223	< 0.001
LF (ms^2^)	128.93 ± 12.38	126.87 ± 11.29	1.440	0.151
HF (ms^2^)	256.23 ± 23.09	135.82 ± 22.13	44.530	< 0.001
LF/HF	0.83 ± 0.23	1.19 ± 0.28	− 11.420	< 0.001
Total EFT (cm^3^)	103.29 ± 12.92	84.29 ± 11.29	13.356	< 0.001
Visfatin (g/ml)	34.87 ± 3.21	23.23 ± 3.19	30.640	< 0.001

#### Analysis of the correlation between the HRV, EFT, and visfatin and AF recurrence

Pearson linear correlation analysis suggested that rMSSD, PNN50, HF, Total EFT and Visfatin were positively correlated with AF Recurrence(r = 0.128, 0.432, 0.399, 0.562, 0.871, *P* = 0.032, 0.021, 0.023, 0.022, 0.032, 0.007), while LF/HF was negatively correlated with AF recurrence(r = − 1.101, *P* = 0.0023) (Table 3).

**Table 3:** Univariate logistic regression analysis of risk factors of AF recurrence post radiofrequency ablation

Variables	β	SE	Wald χ^2^	*P*	OR (95%CI)
rMSSD	0.309	0.201	4.591	0.009	1.401 (1.041–1.737)
PNN50	0.139	0.046	4.349	0.026	1.204 (1.041–1.431)
HF	0.603	0.162	12.568	0.000	1.821 (1.201–2.559)
Total EFT	0.016	0.007	38.558	0.000	1.021 (1.014–1.027)
Visfatin	0.619	0.176	12.584	0.000	1.823 (1.323–2.649)
LF/HF	0.376	0.013	4.338	0.029	1.129 (1.047–1.241)

#### Univariate logistic regression analysis of risk factors of AF recurrence post radiofrequency ablation

The significantly different general variables in the general data and laboratory parameters, rMSSD, PNN50, HF, total EFT, visfatin and LF/HF were used as independent variables, and AF recurrence post radiofrequency ablation was used as dependent variables. Logistic regression analysis revealed that the risk factors of AF recurrence post radiofrequency ablation were rMSSD, PNN50, HF, total EFT, visfatin, and LF/HF.

#### Multivariate logistic regression analysis of risk factors of AF recurrence post radiofrequency ablation

Multivariate logistic regression analysis showed that the risk factors of AF recurrence post radiofrequency ablation were rMSSD, PNN50, HF, total EAT, visfatin, and LF/HF, and the difference was statistically significant (P < 0.05) (Table 4).

**Table 4:** Multivariate logistic regression analysis of risk factors of AF recurrence post radiofrequency ablation

Variables	β	SE	Wald χ^2^	*P*	OR (95%CI)
rMSSD	0.3011	0.203	4.585	0.008	1.398 (1.032–1.741)
PNN50	0.142	0.045	4.341	0.023	1.221 (1.037–1.531)
HF	0.609	0.159	12.572	0.000	1.818 (1.202–2.564)
Total EAT	0.019	0.006	38.562	0.000	1.019 (1.013–1.029)
Visfatin	0.621	0.173	12.579	0.000	1.819 (1.313–2.643)
LF/HF	0.373	0.011	4.332	0.023	1.123 (1.041–1.237)

## Discussion

Atrial fibrillation is occurred mainly due to electrical and structural remodeling of cardiomyocytes [12]. AF poses serious threat to human health, in terms of significantly increasing the incidence of heart failure, embolism, sudden death, and stroke. Radiofrequency ablation has been currently acknowledged as an effective treatment for AF rhythm control and is of greater advantage in maintaining sinus rhythm, increasing the quality of life and improving exercise tolerance than antiarrhythmic medications [13]. Therefore, it has been widely accepted by clinicians and patients, and guidelines in many countries have listed AF as first line therapy [14, 15]. However, considering the high recurrence rate following radiofrequency ablation, repeated radiofrequency ablation therapies not only affects patient compliance but also increases the psychological burden of the patients. Therefore, how to effectively predict AF recurrence post radiofrequency ablation is of great significance and value in guiding clinical treatment.

Abnormality in the autonomic nervous system is a potential trigger of AF onset and a factor in maintaining AF [16]. Changes in the tension of the autonomic nervous system play an important role in the onset, development and persistence of AF. Previous studies have demonstrated that stimulation of the vagus nerve can shorten the refractory duration of the atria and increase the time course dispersion effect of action potential, increasing the trigger ability of AF [17]. Animal studies showed that the joint action of the sympathetic nerve and the vagus nerve predisposes a person to the development of atrial arrhythmia [18]. Copious studies are available to show that abnormality in the autonomic nervous system including the sympathetic nerve, the parasympathetic nerve and cardiac plexus is implicated in the pathogenesis of AF, and selective ablation decreases autonomous nerve control with reduced incidence of spontaneous or induced cardiac arrhythmia [19]. The epicardial fat covers 80% of the cardiac surface and accounts for 20% of the total mass. It is mainly located in the atrioventricular sulcus and interventricular groove [20]. Different from the subcutaneous adipose tissue, it is metabolically active endocrine and paraendocrine organ and secretes massive amounts of anti-inflammatory factors and proinflammatory lipokines, exerting influences on the dynamic equilibrium of energy, glucose and lipid metabolism as well as on the cardiac structure and function. Drabsch et al. [21] reported that EFT could be associated with the onset and development of AF post radiofrequency ablation. Visfatin is a lipid factor produced by the adipose tissue in the internal organs and participates in fibrosis and inflammation. In addition, because of its pro-inflammatory properties and the ability to induce endothelial cell dysfunction, visfatin is also called a cardiovascular disease promoting cytokine. According to Yu et al. [22] visfatin is associated with arterial atherosclerosis, plaque rapture and acute coronary syndrome. Moreover, visfatin has direct effects on cardiomyocytes and the cardiovascular system [23]. Based on current study, the rMSSD, PNN50, HF, total EAT, and visfatin levels in the recurrence group were also higher than that in the non-recurrence group while LF/HF was lower in the recurrence group than the non-recurrence group (*P* < 0.05). It was suggested that patients with AF recurrence post radiofrequency ablation had a longer course of AF with a rising trend of concomitant hypertension, total EAT, and visfatin levels. In addition, among HRV indicators, the rMSSD, PNN50, and HF levels increased while LF/HF decreased, suggesting that the function of the vagus nerve and the sympathetic nerve declined in the patients, specially the function of the vagus nerve.

Currently, the exact mechanisms of AF remain unclear, but researchers have largely studied and found that AF may be a process involving multiple factors including electric conductance abnormality, anatomic anomaly, and inflammation. Chevalier et al. [24] showed that denervation of the parasympathetic nerve is linked to the effectiveness of ablation around the pulmonary vein, and injury of the vagus nerve is conducive to the efficacy pulmonary vein-induced AF ablation. According to the study supported by Patterson et al. [25] the rapid discharge of the components of the sympathetic nerve and the parasympathetic nerve in the pulmonary vein triggers AF, denervated ablation could affect two components of the autonomic nervous system. The rapid discharge of the pulmonary vein is the result of the joint action of neurotransmitters from the sympathetic nerve and the parasympathetic nerve. Therefore, the sympathetic nerve and the vagus nerve have important relationship with AF and its early postprocedural recurrence. It is well established that the denervated vagus nerve is predominant, and the sympathetic nerve also plays an important coordinating role. Akdag et al. [26] and Shin et al. [27] showed that epicardial fat promoted the onset and development of AF by the inflammatory process. Mazurek et al. [28] also demonstrated that glucose metabolism levels in the epicardial fat of AF patients were higher than those in normal subjects, indicating that, compared to normal persons, the epicardial fat of AF patients has higher inflammatory activities, and is able to affect adjacent atrial cardiomyocytes via the paraendocrine pathway to induce local inflammation and promote myocardial remodeling and participate in the onset and development of AF. Marrouche et al. [29] found infiltration of inflammatory cells and fibrosis in the biopsied myocardial tissues of patients with recurrent AF, demonstrating that inflammation is an important component of AF. Visfatin and inflammation are important components of AF. And visfatin is closely related to inflammation, which is able to mediate and instruct cells to produce inflammatory cytokines and lipokines and enhance the oxidative stress of inflammatory reaction via the local interaction between the epicardial tissues and the atria. The current results demonstrated that rMSSD, PNN50, HF, total EFT, and visfatin were positively correlated with AF recurrence post radiofrequency ablation whereas LF/HF were negatively correlated with AF recurrence post radiofrequency ablation (*P* < 0.05). It was suggested that the rMSSD, PNN50, HF, total EAT, visfatin, and LF/HF are highly associated with AF recurrence post radiofrequency ablation patients.

Pappone et al. [30] showed that brief injury of the vagus nerve and reversal of AF-induced electric remodeling could help prevent AF recurrence. Qin et al. [31] found that with the prolongation of the course of AF, the disorder of the autonomous nerve becomes aggravated, and the protection by the vagus nerve is weakened. The lower the value is, the higher the incidence of AF recurrence post radiofrequency ablation. According to Sevinc et al. [32] EFT is independently associated with the onset and development of AF. Moreover, Yang et al. [33] demonstrated that visfatin was an independent risk of AF recurrence post radiofrequency ablation. Consistent with our study, rMSSD, PNN50, HF, total EFT, visfatin, and LF/HF (*P* < 0.05) were also risk factors responsible for AF recurrence post radiofrequency ablation. And our multivariate logistic regression analysis further confirmed the earlier results.

Nevertheless, this study had the following limitations: First, the overall sample size is relatively small. Second, because of the retrospective nature of this study, all patients enrolled in this study were from a single center. Future multicenter prospective studies are needed to confirm the current findings.

As exhibited with our preliminary results, higher HRV and greater EFT appear to be the risk factors responsible for AF recurrence. Hence it is necessary to detect HRV of patients after radiofrequency ablation, in order to proactively prevent AF recurrence.

## Data Availability

The datasets generated and analyzed during the current study are available from the corresponding author on reasonable request.

## Abbreviations

EFT: Epicardial fat thickness
HRV: Heart rate variability
HF: High frequency
LF: Low frequency
NN: Normal-to-normal
PNN50: Percentage of adjacent NN intervals that differed from each other by more than 50 ms
RMSSD: Root mean square standard deviation of adjacent R-R intervals
ROI: Region of interest
SDNN: Standard deviation of the normal-to-normal
SDANN: Standard deviation of the average normal-to-normal
